# Comparative clinical study testing the effectiveness of school based oral health education using experiential learning or traditional lecturing in 10 year-old children

**DOI:** 10.1186/s12903-015-0036-4

**Published:** 2015-04-28

**Authors:** Matina V Angelopoulou, Katerina Kavvadia, Konstantina Taoufik, Constantine J Oulis

**Affiliations:** 1 Division of Pediatric Dentistry, Marquette University, School of Dentistry, 1801 W Wisconsin, 53233 Milwaukee, WI USA; 2 Department of Paediatric Dentistry, Dental School, University of Athens, Thivon 2 Goudi, 11527 Athens, Greece; 3 Private Practice, Athens, Greece

**Keywords:** Oral health education, Experiential learning, Primary school, Oral hygiene, Traditional lecturing

## Abstract

**Background:**

School based oral health education through traditional lecturing has been found successful only in improving oral health knowledge, while has low effectiveness in oral hygiene and gingival health. The aim of this study was to evaluate the effectiveness of experiential learning (EL) oral health education to traditional lecturing (TL), on enhancing oral health knowledge, attitude and behavior as well as oral hygiene, gingival health and caries of 10-year-old children.

**Methods:**

Eighty-four children were recruited for the EL and 100 for the TL group from 3 locations in Greece. Data regarding oral health knowledge, attitude and behavior were collected via questionnaires. Data regarding dental plaque, gingivitis and caries were collected by clinical examination. The evaluation using questionnaires and clinical examination was assessed at baseline and 6 and 18 months afterwards. Two calibrated pediatric dentists examined the students using a periodontal probe and artificial light. Modified hygiene index (HI) was used for dental plaque recording, the simplified gingival index (GI-S) was used for gingivitis and DMFT, based on BASCD criteria, for dental caries. Based on a dedicated manual, the teacher applied in the classroom the oral health educational program using EL.

**Results:**

EL group had statistically significant better hygiene than the TL at 6 months (p < 0.05). Within the same group, both groups had enhanced oral health knowledge at 6 and 18 months (p < 0.05) and improved oral health behavior (p > 0.05) and attitude (p > 0.05) at 6 months in comparison to baseline.

**Conclusion:**

EL program was found more successful than TL in oral hygiene improvement. Both oral health education programs improved the oral health knowledge, attitude and behavior of children.

**Trial registration:**

ClinicalTrials.gov (NCT02320162).

**Electronic supplementary material:**

The online version of this article (doi:10.1186/s12903-015-0036-4) contains supplementary material, which is available to authorized users.

## Background

Dental professionals globally struggle to improve public oral health using oral health education which enhances oral health literacy and aims to behavioral change [1]. Especially for children, school based health programs are the most common, since such programs can benefit a wide group of children with extremely low cost [2-4]. School-based oral health education has been found effective in improving oral hygiene, oral health knowledge and behavior [5-11].

Results of a recent epidemiological study in Greece, regarding 12 years-old children oral health, demonstrated a 78,2% of average or poor oral hygiene and a 41,5% with gingivitis [12]. The above findings show the need to educate children prior to the age of 12 in oral health issues.

Up to date, the traditional didactic method of the lecture (verbal or videotaped) is the most frequently used oral health educational approach, showing oral health knowledge [1,10] improvement but insufficient in changing oral health behavior and attitude [13-15].

Experiential learning is a educational approach where learning comes through experience [16,17]. It has been used effectively in enhancing knowledge and improving attitude in health education [18,19] and recently was introduced in oral health education with promising results [5-8,20]. However, these programs have used isolated experiential learning techniques lacking the benefit of a comprehensive experiential learning program [5-8,20]. Furthermore, all the studies reported have tested the short-term effect of these programs and in a limited sample [5-8,20].

The aim of this study was to evaluate the effectiveness of experiential learning (EL) or traditional lecturing (TL) school-based oral health education on improving the oral health knowledge, attitude and behavior as well as oral hygiene, gingival health and caries of 10-year-old children in Greece.

## Methods

### Study design

This was a 2-arm parallel-group prospective clinical trial, between two oral health education interventions, experiential learning (EL) and traditional lecturing (TL). TL group had only a lecture on oral health issues by a dentist, acting as the control group whereas EL group received additionally oral health education by their teacher using the program’s manual. Oral health knowledge, attitude and behavior as well as clinical parameters were evaluated via questionnaire and clinical examination at baseline, 6 and 18 months. This study was in full accordance with the World Medical Association Declaration of Helsinki and had the approval of the University of Athens Ethical Committee and the Greek Ministry of Education (30.10.09, No126516/Γ7). This clinical trial was part of a larger study registered in the ClinicalTrials.gov (ID: NCT02320162) under the name “School-based Oral Health Education Program Using Experiential Learning”.

### Sample

Sample recruitment was clustered by school. Schools contributing students for the trial were of rural, low socioeconomic urban or high socioeconomic urban areas as determined by the Hellenic Statistical Authority.

*Inclusion criteria* for sample recruitment were the following: *a)* to be attending the 4th grade of primary school (approximately ten years old) with non contributory medical history, *b)* EL group students to be attending schools, that had teachers previously trained in experiential learning by the Greek Ministry of Education. Trained teachers were confirmed by a record of participants in past educational seminars and were asked to apply for participation in the experiential learning oral health education program. Final assortment was made on the basis of “first come, first served”, *c)* TL group students to be attending schools of the same area as the EL group in order to match their socioeconomic level and grade level, *d)* parental written informed consent from all participants.

Initially 274 students from 7 public schools around Greece were asked to participate in the study. Parents of 210 students gave informed consent for student’s participation in the study and finally 184 students participated in re-evaluation. Sample size diminished through the recall visits due to students having moved to other schools or being absent the day of the exam. Thus the final sample consisted of 184 ten years old students.

### Interventions

Experiential Learning. A teacher’s manual was developed for the EL group. Participating teachers received a seminar on oral health education, experiential learning methods and how to use the program’s manual. Questionnaires were filled out by children at schools regarding their oral health knowledge, habits and attitude. A dentist’s lecture regarding oral health issues was given in the classroom at baseline. Oral examination was carried out in the classroom for evaluation of plaque, gingival health and caries and toothbrushes and fluoride toothpastes for home use were distributed to all children. Then, teachers familiar with EL teaching technique implemented the EL program for 3 months, according to a preset course using the program’s manual (book & CD available in www.oral-health.gr). Firstly, the teacher introduced the aim and the procedure of the oral health education program and a discussion concerning students’ attitude and feelings regarding oral health was initiated using brainstorming. The children were then divided in working groups and were given specific oral health projects. Extramural visits e.g. to the dentist, to the vet, to the pharmacy or supermarket were also carried out. Within the classroom each group, carried out a presentation of their project’s outcome using different forms such as theatrical play, posters, songs, crafts, role playing etc. Then, the teacher stimulated a discussion in the classroom in order to answer possible questions and give the opportunity to children to expose their feelings and experientially realize the significance of oral health. At the end of this session, a dentist visited the classroom to demonstrate oral hygiene and respond to possible scientific questions. Moreover, children’s project products such as t-shirts, posters, games, crafts, songs and theatrical plays were uploaded in the program’s web-site (www.oral-health.gr) to motivate other students and teachers for the program. Student’s re-evaluation was carried out after the completion of the program via questionnaires and clinical examination at 6 and 18 months to the baseline.

Traditional Lecturing. TL group followed the same procedure concerning data collection and evaluation. However, children of the TL group attended only the power point presentation on oral health issues given by the dentist at school without having any further education from their teacher. Teachers in the TL schools did not participate in the seminar for experiential oral health education.

### Procedure

#### Questionnaires

Data regarding children’s oral health knowledge, attitude and behavior were collected via questionnaires at baseline and at 6 and 18 months. The questionnaire included questions written in lay terms and in the children’s native language in order to be easily apprehended by children of this age. The questionnaire was distributed to 20 children of the same age prior to their application for validation. In some questions the option to select more than one answer was given to the student if more than one answer applied to them ex. twice a day brushing. The educational level of both parents was reported in a questionnaire dedicated to parents. A version of the questionnaire translated in English is presented in Additional file 1: Table S1.

Questions were graded and a total score was calculated for knowledge (questions 1–8), behavior (questions 9–19) and attitude (questions 20–22) by adding the score of each question related to each category.

#### Clinical examination

Two calibrated blinded pediatric dentists clinically examined all children at baseline and at 6 and 18 months in their classroom using a mirror, a periodontal probe and artificial light. The variables recorded were the following: a) dental plaque using a modification of hygiene index (HI) of Lindhe, that does not use a disclosing agent [21], b) gingivitis using the simplified gingival index (GI-S) [21] and c) dental caries (DMFT), according to the diagnostic criteria of the British Association of Community Dentistry, BASCD [22].

More specifically, HI and GI-S were recorded measuring dental plaque and bleeding as present or absent on the distal, mesial and buccal surfaces of the permanent molars and anterior teeth. All children presenting bleeding on probing were diagnosed with gingivitis.

Caries was measured only at baseline and 18 months since caries progressing is slow and no differences were expected to be found 6 months after the baseline examination.

### Outcome measures

The primary outcome was the effect of experiential learning on the reduction of dental plaque, gingivitis and caries as compared to the traditional lecturing method.

Secondary outcomes were the improvement of oral health knowledge, behavior and attitude, as reported from the questionnaires in children following the EL program compared to those who did not (TL program).

### Statistical analysis

Data were reported descriptively by calculating Median and Interquartile Range (IQR) or Mean and Standard Deviation (SD). For caries index, intraclass correlation coefficient (ICC) in 20 patients was used to test inter examiner reliability. Results from the questionnaires and clinical examination were statistically analyzed to identify differences, between and within the comparative groups.

Non parametric tests (Mann Whitney *U*-test and Kruskal-Wallis test) were used for the statistical comparison of quantitative variables among the same group (EL or TL). Analysis between the two intervention groups (EL vs TL) was based on fractional logit models for repeated measurements estimated through generalized estimating equations (GEE) [23,24]. Adjustments for demographic characteristics (gender, location, parents educational level) were made in order to take into account the distribution of the dependent variables and the correlation between initial and final measurements on the same individual. Statistical significant differences were investigated at the level of p < 0.05 using Stata 10.2 software (Stata Corp., TX USA).

## Results

Eight-four school children were allocated to the EL group and 100 to the TL group. Power analysis was 82% at *α* = 0.05. Sample’s socioeconomic data are presented in Table 1.

**Table 1:** **Demographic data for the EL and TL groups and for the total sample**

	EL	TL	Total sample
	N		
**Gender**			
*Male*	45 (54%)	55 (55%)	100 (54%)
*Female*	39 (46%)	45 (45%)	84 (46%)
**Location**			
*Low Urban*	18 (21%)	22 (22%)	40 (22%)
*Rural*	55 (66%)	59 (59%)	114 (62%)
*High Urban*	11 (13%)	19 (19%)	30 (16%)
***Total***	84	100	184

At baseline the two intervention groups did not present any statistical significant differences. Descriptive results at baseline for each group are presented in Table 2. The inter examiners reliability for assessing caries had an ICC of 0.89.

**Table 2:** **Questionnaire scores and clinical examination data at baseline, 6 and 18 months**

		EL			TL		
		Baseline	6 months	18 months	Baseline	6 months	18 months
	*N*	84	82	67	100	87	76
		Median (IQR)					
**Questionnaire**	Knowledge (%)	71.0 (67.7, 74.0)	75.4 (71.7, 78.8)*	81.7 (77.8, 85.0)*	66.9 (62.7, 70.8)	78.7 (74.9, 82.1)*	78.9 (75.2, 82.2)*
	Behavior (%)	71.7 (68.3, 74.9)	72.0 (68.8, 74.9)	69.3 (66.0, 72.4)	69.0 (65.9, 71.8)	71.2 (68.5, 73.8)	68.6 (65.9, 71.2)
	Attitude (%)	80.8 (75.9, 84.8)	83.3 (78.7, 87.1)	81.6 (76.8, 85.6)	81.8 (77.5, 85.5)	83.4 (78.8, 87.2)	79.1 (74.7, 82.9)
**Clinical examination**	Hygiene Index (%)	64.6 (38.0, 83.3)	69.4 (46.7, 83.3)	55.6 (29.2, 79.2)	57.7 (30.6, 80.6)	50.0 (29.2, 76.3)	66.7 (37.6, 83.3)
	Gingival Index (%)	31.2 (19.4, 41.7)	33.3 (22.2, 47.2)	22.2 (12.5, 43.8)	34.4 (17.7, 48.7)	36.1 (22.2, 52.6)	26.0 (8.3, 41.1)*
	DMFT (Mean, SD)	0.77 (1.13)	-	1.01 (1.45)*	0.55 (1.16)	-	0.87 (1.30)*

Statistical significant differences from the baseline among the same intervention group EL or TL at 6 and 18 months were calculated using Mann Whitney *U*-test or Kruskal-Wallis test and are marked with *.

Descriptive results from the questionnaire and the clinical examination at 6 and 18 months as well as differences detected within the same group are presented in Table 2. Within the same group, oral health knowledge improved in both groups in a statistical significant level at 6 months (EL group p = 0.022, TL group p = 0.001) and 18 months (EL group p = 0.001, TL group p = 0.001) (Table 2). Also, within the same group oral health behavior improved in both groups at 6 months but decreased at 18 months and the changes were not significant for neither of the groups (Table 2). The same trend was noted for the oral health attitude both at 6 and 18 months (Table 2). Regarding the clinical results, oral hygiene improved for the EL group at 6 months while it was decreased for the TL group but the changes were non statistical different within the same group. Gingivitis increased within the same group for both groups at 6 months in a non statistical significant level and then decreased at 18 months (Table 2). The difference at 18 months was statistically significantly only for the TL group (p = 0.004) (Table 2). Concerning caries index, it significantly increased in both groups (EL group p = 0.013, TL group p = 0.010) at 18 months.

Statistical significant differences detected between the two groups are presented in Table 3. Regarding the data from the questionnaire, no statistical significant differences were noted between the two groups (Table 3). Regarding the clinical data, comparing the two interventions, EL group had statistically significant improved hygiene index than the TL group at 6 months (p = 0.015) (Table 3). However the difference was not statistically significant between the groups at 18 months (Table 3). Gingival index and DMFT index did not differ between the two intervention groups (Table 3).

**Table 3:** **Comparison between the EL and TL intervention group at 6 and 18 months for the different parameters evaluated**

	EL vs TL p-value	
	6 months	18 months
Knowledge	0.143	0.061
Behavior	0.644	0.692
Attitude	0.911	0.268
Hygiene Index	0.015*	0.173
Gingival Index	0.519	0.745
DMFT	-	0.601

Statistical significant differences were calculated using generalized estimationg equations-GEE and are marked with *.

## Discussion

Results of the present study suggest that experiential learning is an effective school based oral health education method for short term improvement of oral hygiene in primary school children.

School based oral health education programs in the past using traditional lecturing had been found effective in improving knowledge [13]. However, students were not able to positively impact their oral health behavior [1,10]. Also, usually a dentist or a dental hygienist applies the oral health education program at the school [6,10,25-27]. This is the first study to use experiential learning for a comprehensive school based oral health education program. Additionally, this program exploits the teacher who is most suited to implement such a program since he has daily contact with the students and is experienced in teaching skills [28-30]. In order for teachers to be able to apply successfully an experiential learning program, related training is necessary [31-33], thus, a day seminar was given to the participating teachers. In the present study in order to assure that experiential learning will be effectively implemented, a convenience sample was selected choosing schools according to the teachers experience in experiential learning techniques and volunteer offer to participate.

The target group for the specific oral health education program was the primary school children. The high prevalence of gingivitis found in a recent epidemiological study in 12 year old children in Greece made it imperative to enhance oral health education at an earlier age in order to improve plaque removal and monitor gingivitis in later years [12]. Also, 10 year old children were selected because experiential learning requires students that can do logic thoughts, can work together in teams, can realize the cause-result interaction and explore everything. Younger children possibly would not be able to present those skills and experiential learning would be ineffective [17].

Evaluation of the present program was quantitative using a questionnaire and clinical examination. Quantitative results, in an oral health education program based in experiential learning, have been assessed only in one study previously [30]. However, that program was not comprehensive concerning experiential learning process and was evaluated only 6 months after intervention. The clinical indexes of hygiene and gingivitis were chosen because they are simple, easy and objective since they are based on the presence or absence of bleeding and dental plaque despite examiner’s estimation [21]. Dental caries were assessed at the cavitation level using the BASCD criteria and estimating the DMFT index in order to be able to compare the results with other studies. The index for caries in the primary dentition (dmft) was also recorded, however, it was not taken into consideration since many of the primary teeth were lost during the 18 months and it would bias the results. For this reason, only the permanent teeth that were present in all clinical examinations were taken into account for the calculation of the DMFT index.

Statistical analysis was based on non-parametric tests since data did not have a normal distribution. However, further analysis was based in GEE models that are reliable even in cases of high data deviation [24].

Results of the present study suggest that knowledge improved in both intervention groups. However, knowledge score for the EL group showed a trend of further improvement at 18 months whereas TL group’s knowledge remained almost the same. Enhanced oral health knowledge has been stated previously even when applying traditional lecturing [5-7,10,26,34]. Previous experiential learning studies suggested that knowledge improves more when this method is being used [19,32,35-38].

Both interventions were found effective in improving oral health behavior and attitude. This finding is in accordance with other studies that found that oral health behavior and attitude of primary school children to temporary improve regardless of the educational approach used [8,11,26,39]. Some other studies, found no improvement when using the traditional methods [15,40] in contrast to the finding of the present study; however, neither the present oral health education program managed to sustain the improved oral health attitude and behavior in the long-term. These findings, as suggested in the past, prove that oral health education should be repeated with either method in order to maintain its positive results longitudinally [40,41].

Regarding clinical data, experiential learning group showed improved oral hygiene as previously found when using experiential learning techniques [5,6,8]. However, the improvement was temporary which is in accordance with the findings of other studies [5,6,8-10,15,34,39,42,43]. In the traditional lecturing group, no statistically significant improvement has been found even for the short term, suggesting that experiential learning is a more effective method for oral hygiene instruction.

Concerning gingival health no improvement was detected at 6 months either for the EL or the TL group. Slight improvement in gingival health for both groups at 18 months may have been influenced by the normal age maturation and the transition from mixed to the permanent dentition. Children were first examined at the age of 10 years old, around the time of the eruption of the permanent canine and premolars that may cause gingivitis since children neglect brushing in the fear of pain due to the mobility of loose teeth. When they were re-examined at the age of 11.5 years old they might have outgrown the eruption gingivitis, were more mature, and had started being interested more in their self image [44,45]. Literature is inconclusive on the above since some studies report improved gingival health [5,10,27,42] while others don’t [40,43].

Caries increased in both groups and did not differ 18 months after the intervention. These results are in accordance with previous findings from traditional oral health education programs [9,34]. However, 18 months may be a short time to detect differences between groups since caries progress slowly.

Experiential learning has been proven more effective in adolescents [46] compared to primary school children, as expected since experiential learning was developed as an adult educational method [31,47]. This difference may be attributed to primary school children being more influenced by their parents [48-51] and adolescents being more aware of taking care of themselves in order to attract the opposite gender [44,45]. In addition, primary and secondary schools follow a different curriculum that may have influenced the application of the program. As mentioned above, experiential learning programs’ effectiveness is influenced by the environment and the educator [3,16,31-33].

Limitation of the present study is the convenience sample which did not represent the Greek population. Also, sample size drop outs due to students moving, might have influenced the result at the 18 month evaluation. Another confounding factor might be the alterations between schools during the implementation of the experiential program. Although the program was based on the dedicated manual and the same education was given to all participating teachers, still differences might have occurred due to the dynamic interaction between the teacher and specific students group.

Results from this study promoted the application of this program nationally through the Ministry of Education. In the future, this program could be reinforced with parents’ involvement in the educational process and other preventive measures such as tooth brushing and fluoride treatment in order to enhance its effect. Also this program could be used in earlier age groups too, which may contribute to better results in the later years.

## Conclusions

Results of the present study suggest that experiential learning can be used as a method for oral health education programs in order to obtain better oral hygiene; however it should be repeated frequently.

## Additional file

Additional file 1: Table S1. Implemented questionnaire regarding children’s oral health knowledge, attitude and behavior distributed at baseline and at 6 and 18 months after the intervention.

## Abbreviations

EL: Experiential learning
TL: Traditional lecturing
HI: Hygiene index
GI-S: Gingival index simplified
BASCD: British Association for the Study of Community Dentistry
DMFT/DT: Decayed, missing, filled teeth
CD: Compact disc
ICC: Intraclass correlation coefficient
SD: Standard deviation
IQR: InterQuartile range
GEE: Generalized estimating equations
