# Supplementary material for: Comparative clinical study testing the effectiveness of school based oral health education using experiential learning or traditional lecturing in 10 year-old children
Source: BMC Oral Health. 2015 Apr 28;15:51. doi: 10.1186/s12903-015-0036-4 (PMC4415446; doi:10.1186/s12903-015-0036-4)
Supplement: Additional file 1: Table S1. — Implemented questionnaire regarding children’s oral health knowledge, attitude and behavior distributed at baseline and at 6 and 18 months after the intervention. [file 12903_2015_36_MOESM1_ESM.doc]

Additional file 1: Table S1. Implemented questionnaire regarding children’s oral health knowledge, attitude and behavior distributed at baseline and at 6 and 18 months after the intervention.

| **QUESTIONNAIRE** | |
| --- | --- |
| Code: ...................................... Date:........................ Gender: Boy 🗖 Girl 🗖  Father’s occupation: .............................................. Mother’s occupation: ...............................................  Father’s educational level: a)elementary b) high school c) university Mother’s educational level: a)elementary b) high school c) university | |
| 1. **How often should we brush our teeth?**   a) Once a day in the morning  b) Once a day in the evening  c) After each meal  d) When we remember  e) I don’t know  **2. We use dental floss to clean:**  a) the lip  b) the areas between the teeth  c) the chewing surfaces of the teeth  d) I don’t know  **3. When should we visit the dentist?**  a) Whenever we hurt or have a problem  b) At least once a year for check-up  c) When entering primary school  d) When entering high school  **4. What is the cause of caries?**  a) Dirty hands  b) Frequent consumption of sweets and soda  c) Frequent tooth brushing  d) The meat  **5. Dental plaque is:**  a) Membrane of bacteria on teeth  b) Caries  c) Red mass on gums  d) I don’t know  6**. What is the first sign of gingivitis?**  a) Oral ulcers  b) White gums  c) Swollen gums that bleed  d) I don’t know  7**. Which foods harm our teeth?** *(choices may be more than one)*  a) Chocolate milk  b)Yogurt with honey  c) Sweets without chocolate  d) Juice  e) Fruits  f) Carrots & cabbage  g) Fresh juice  **8. When is better to eat a sweet?**  a) After meal  b) Between meals  c) Before going to bed  d) I don’t know  **9. How often do you brush your teeth daily?**  a) After each main meal  b) Once a day in the morning  c) Once a day in the evening  d) Not every day  **10. Do you use toothpaste?**  a) Yes  b) No  **11. Do you use dental floss?**  a) Yes  b) No | **12. Do your gums bleed?**  a) Yes  b) No  **13.If your teeth are dirty:**  a) You don’t care  b) It bothers you a little  c) It bothers you a lot  **14. You drink your milk:**  a) Plain  b) With sugar  c) With honey  d) With chocolate  e) I don’t drink milk  **15. Have you ever visited a dentist?**  a) Yes  b) No  **16. Why did you first visit the dentist?**  a) Because of a toothache  b) To fix my teeth  c) To check my teeth  d) I don’t know/ remember  **17. Did you visit the dentist in the last 12 months?**  a) No I didn’t  b) Once  c) Many times  d) I don’t know/ remember  **18. Did you ever had fluoride treatment at the dentist?**  a) Yes  b) No  c) I don’t know  **19. Did the dentist inform you how to take care of your mouth?**  a) Yes  b) No  **20. How do you feel when you go to the dentist?**  a) Indifferent  b) Unhappy  c)Happy  **21. Why do you think that your peers do not brush their teeth** *(choices may be more than one)*  a) They don’t know how to do it correctly  b) They forget  c) They are bored  d) They don’t like the flavor of the toothpaste  e) Their parents do not remind them  f) They do not know the importance of brushing on their health and image  g)Other  **22. Do you believe that your oral health may impact your general health?**  a) Not at all  b) Small  c) In between small and high  d) High  e) Too high |
